# Photobiomodulation—Underlying Mechanism and Clinical Applications

**DOI:** 10.3390/jcm9061724

**Published:** 2020-06-03

**Authors:** Claudia Dompe, Lisa Moncrieff, Jacek Matys, Kinga Grzech-Leśniak, Ievgeniia Kocherova, Artur Bryja, Małgorzata Bruska, Marzena Dominiak, Paul Mozdziak, Tarcio Hiroshi Ishimine Skiba, Jamil A. Shibli, Ana Angelova Volponi, Bartosz Kempisty, Marta Dyszkiewicz-Konwińska

**Affiliations:** 1 The School of Medicine, Medical Sciences and Nutrition, Aberdeen University, Aberdeen AB25 2ZD, UK; claudia.dompe.16@abdn.ac.uk (C.D.); l.moncrieff.16@abdn.ac.uk (L.M.); 2 Department of Histology and Embryology, Poznan University of Medical Sciences, 60-781 Poznań, Poland; bkempisty@ump.edu.pl; 3 Laser Laboratory at Dental Surgery Department, Medical University of Wroclaw, 50-425 Wroclaw, Poland; jacek.matys@wp.pl (J.M.); kgl@periocare.pl (K.G.-L.); marzena.dominiak@umed.wroc.pl (M.D.); 4 Department of Periodontics, School of Dentistry Virginia Commonwealth University, VCU, Richmond, VA 23298, USA; 5 Department of Anatomy, Poznan University of Medical Sciences, 60-781 Poznan, Poland; ikocherova@ump.edu.pl (I.K.); abryja@ump.edu.pl (A.B.); mbruska@ump.edu.pl (M.B.); 6 Physiology Graduate Program, North Carolina State University, Raleigh, NC 27695, USA; pemozdzi@ncsu.edu; 7 Department of Periodontology and Oral Implantology, University of Guarulhos, Guarulhos 07030-010, Brazil; tarciohiroshi@gmail.com (T.H.I.S.); jshibli@ung.br (J.A.S.); 8 Centre for Craniofacial and Regenerative Biology, Dental Institute, King’s College London, London WC2R2LS, UK; ana.angelova@kcl.ac.uk; 9 Center of Assisted Reproduction, Department of Obstetrics and Gynecology, University Hospital and Masaryk University, 601 77 Brno, Czech Republic; 10 Department of Veterinary Surgery, Institute of Veterinary Medicine, Nicolaus Copernicus University in Torun, 87-100 Torun, Poland; 11 Department of Biomaterials and Experimental Dentistry, Poznan University of Medical Sciences, 61-701 Poznan, Poland

**Keywords:** laser, low-level laser therapy, photobiomodulation, stem cells, tissue regeneration

## Abstract

The purpose of this study is to explore the possibilities for the application of laser therapy in medicine and dentistry by analyzing lasers’ underlying mechanism of action on different cells, with a special focus on stem cells and mechanisms of repair. The interest in the application of laser therapy in medicine and dentistry has remarkably increased in the last decade. There are different types of lasers available and their usage is well defined by different parameters, such as: wavelength, energy density, power output, and duration of radiation. Laser irradiation can induce a photobiomodulatory (PBM) effect on cells and tissues, contributing to a directed modulation of cell behaviors, enhancing the processes of tissue repair. Photobiomodulation (PBM), also known as low-level laser therapy (LLLT), can induce cell proliferation and enhance stem cell differentiation. Laser therapy is a non-invasive method that contributes to pain relief and reduces inflammation, parallel to the enhanced healing and tissue repair processes. The application of these properties was employed and observed in the treatment of various diseases and conditions, such as diabetes, brain injury, spinal cord damage, dermatological conditions, oral irritation, and in different areas of dentistry.

## 1. Introduction

The role of light in biological systems and processes can be observed clearly in our sleep–wake cycles, circadian rhythms, and in the absorption of vitamins. The introduction of lasers, as sources of amplified, stimulated emission of radiation, gave researchers an opportunity to obtain and use high-powered light (at specific wavelengths) in biology, creating a new perspective for its application in healing and tissue engineering. In medicine and dentistry, lasers used in phototherapy and included in photobiomodulation (PBM) are low-level lasers (class III) which are defined with an output power of 500 mW, and there are also high-level lasers (class IV) with a power output of 500 mW or more [1,2].

At the same time, the research focusing on stem cells and their properties contributes to better understanding of the repair and regeneration mechanisms, based on properties of stem cells such as self-renewal and ability of multilineage differentiation [3].

The effect of PBM in accelerating the healing process was introduced in the 1960s by Endre Mester and National Aeronautics and Space Administration (NASA) researchers used it for enhancing the healing processes in space [4]

Laser and LED light induce a photobiomodulation (PBM) effect which is used to accelerate healing, as it increases cell viability by stimulating the mitochondrial and cell membrane photoreceptors synthesis of ATP. This process can be used in promoting the proliferation rate of osteoblasts, allowing the development of new clinical approaches where the influence of laser irradiation will be cross-linked with the knowledge of stem cell behaviour and directed manipulation toward accelerated bone repair [5,6,7].

## 2. Bio-Modulation of Human Cells and Tissues by Using Lasers

The use of laser therapy, through the technique of photobiomodulation (PBM), is yet to be part of standardized treatment for tissue repair and regeneration, due to a lack of understanding of how the mechanism works on cells and tissues [8]. Also known as low-level laser light therapy (LLLT), as it is based on the use of lasers (or LED light) that produce low intensity light, PBM is non-invasive and has been shown to decrease inflammation and provide pain relief [9]. Therefore, treatments of tendinopathies, nerve injuries, osteoarthritis, and wound healing could all benefit from the use of laser therapy on tissues [8,10]. Mild side-effects from the therapy can include cutaneous irritation, itching, and redness, which are not very harmful and do not result in a rise in temperature of the target tissue [11].

A key area of interest concerning PBM is its effect on susceptible stem cells, progenitor cells, and its potential in enhancing differentiation, which in turn improves the healing rate of tissues [8]. Multiple studies have reported that stem cell proliferation is improved by photobiomodulation, such as gingival fibroblasts [1], dental pulp stem cells extracted from permanent teeth [2], exfoliated deciduous teeth [3,4], in addition to mesenchymal stem cells derived from bone marrow or adipose tissue [5,6]. Liao et al. concluded in their research on the effect of PBM on epidermal stem cells that cell migration, in addition to proliferation, was also improved, and that there was no observed effect on differentiation [7]. In one study, a number of dental derived mesenchymal stem cell markers (including STRO-1, CD90, CD117, and CD44) decreased after PBM, suggesting differentiation was promoted [8]. However, Ferreira et al. found contrary evidence in their study when they used PBM on dental pulp stem cells, which highlights the pattern of difficult to replicate studies in laser research [9].

The effectiveness of PBM on the target tissue is dependent on the parameters used such as light source, wavelength, energy density, light pulse structure, and the duration of the laser application [12]. Epigenetic mechanisms, which are regulated by environmental cues, are also affected by laser treatment [11]. Red light or near-infrared light (NIR) are the most commonly used wavelengths in PBM (600–1100 nm) [13]. In longer wavelengths, 1064 nm is often chosen for studies and applications [14,15]. However, Wang et al. discovered that blue (420 nm) and green (540 nm) light enhanced the differentiation of human adipose-derived stem cells (hADSCs), highlighting the possibilities of translating this knowledge into novel future therapeutic approaches [16]. The effectiveness of different types of laser and wavelength will be further presented in the paper.

Although PBM has been the focus of many research groups in recent years, shedding light on the underlying mechanisms of its action may lead to a better understanding of the effects of PBM on cells and their various benefits for improved healing. For example, one theory states that PBM reacts with target cells photochemically [17]. Mitochondria contain chromophores which absorb photons from PBM. The primary chromophore to absorb red light is the enzyme cytochrome c oxidase, which is located at unit IV of the mitochondrial respiratory chain, resulting in the activity of various molecules such as nitric oxide (NO), ATP, calcium ions, reactive oxygen species (ROS), and numerous other signaling molecules [13]. It is thought that glycolysis and ATP production are promoted due to PBM stimulating electrons in chromophores to move from higher energy orbits, and then electron carriers (such as the chromophore cytochrome c oxidase) deliver these electrons to their ultimate electron acceptors whilst a proton gradient is made, in addition to creating a proton gradient that increases ATP production. Furthermore, various transcription factors are switched on by PBM [8,11,18,19]. PBM also encourages the production of ROS in a similar fashion as photodynamic therapy (PDT). The difference between the two phototherapy groups is that while PBM is used to improve wound healing or as pain relief, PDT is a combination of light and photo-sensitive drugs targeted at chromophores to destroy microbes or oncogenic cells [20,21]

A theory for the mechanism of action for PBM on cells isphoton radiation posits either directly or indirectly targeting DNA and the genome pool. Free radicals (including ROS) are produced on the impact of indirect, low laser radiation. High levels of ROS are known to be cytotoxic, leading to multiple signaling cascades being interrupted [22]. However, in low levels, ROS can be beneficial to red blood cells and induce apoptosis in breast cancer MCF-4 and pre-osteoblastic MC3T3 cells, by acting as secondary messengers to various signaling pathways [12,23]. ROS can generate normal metabolism in the production of ATP synthesis and regulate proteins that are affected by redox reactions and involved in proliferation and differentiation [11,23]. Cells will then migrate to repair the damage that has occurred from the laser treatment. Together with cytokines and growth factors, ROS can help tissue recovery as it induces the transport of myogenic precursor cells to the damaged site [22]. Cyclooxygenase (COX) enzymes are involved in the normal run of the membrane potential of the mitochondria and collect ROS to ensure this. These enzymes stop the production of unnecessary ROS when the mitochondrial membrane potential has decreased, which can be due to various factors caused by toxic environments, such as oxidative stress, damaged neurons, or the suppression of electron transport. While ROS production is stopped, COX will act to restore the mitochondrial potential back to normal levels [11]. Regulation of ROS levels is important, as it affects several signaling pathways responsible for the development and proliferation of stem cells [11].

**Figure 1:**
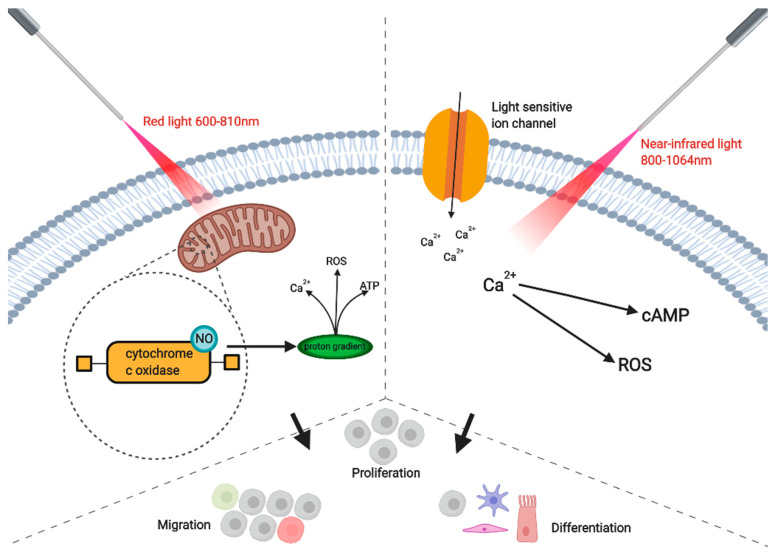
The application of red light (600–810 nm) is absorbed by the enzyme cytochrome c oxidase, which is located in the unit IV respiratory chain of the mitochondria. Nitric oxide (NO) is then displaced and activates the enzyme and this leads to a proton gradient. Consequently, calcium ions (Ca^2+^), reactive oxygen species (ROS), and ATP production levels are increased. On the other hand, the application of near-infrared light (810–1064 nm) activates light-sensitive ion channels, and increases the levels of Ca^2+^. ROS and cyclic AMP (cAMP)then interact with the calcium ions. All of these activities increase cell differentiation, proliferation and migration, among other things. Created with BioRender.

Some research has suggested that laser irradiation in the region of red light increases activity in the plasma membrane of cells [12]. Low biochemical activity occurs in wavelengths in the range of 700–770 nm. The optimum wavelength in treatment is usually considered to be 810 nm [11]. However, wavelengths that range to 950 nm are necessary to reach cutaneous tissue sites and farther below the tissue. Wang et al. state that 810 nm can be absorbed by the chromophore cytochrome c oxidase and other chromophores to improve mitochondrial activity [24]. This primary chromophore has two bands which gives it the 600–810 range. Cytochrome c oxidase is the main chromophore to absorb red light, but different molecules at higher wavelengths are thought to also absorb the higher wavelengths out of range for cytochrome c oxidase [16]. This theory is thought to involve light and heat-gated channels, such as members of the transient receptor potential (TRP) family with the photon absorption range of 980–1064 nm [13]. One theory as to how cytochrome c oxidase works posits that inhibitory NO is displaced by irradiation, and then binds to the copper and heme centers of the chromophore to activate it. The activity of cytochrome c oxidase increases the build-up of mitochondria activity, which leads to a larger production of ATP. Alternatively, the activity of the chromophore perhaps blocks oxygen’s access to the active site of cytochrome c oxidase [13]. Differentiation of stem cells is enhanced, possibly due to the change of priorities in the now ATP rich cell, shifting from glycolysis to oxidative phosphorylation, and this could be considered as a metabolic switch that is a known key factor in osteogenesis. Other by-products of PBM, especially ROS, are associated with enhanced differentiation. Then, the various series of secondary actions occur (Figure 1). One of these secondary actions is Ca^2+^ entering cells via the light-sensitive gated ion channels, which results in various reactions with ROS, NO, and cyclic AMP (cAMP), which then results in the activity of transcription factors [9]. Besides red light, Wang et al.’s use of blue and green light suggests that, at lower wavelengths, light-gated ion channels could be stimulated. A different chromophore could also be activated by blue and green light, such as one present in channel rhodopsin [16].

## 3. Cellular, Sub-Cellular, Morphological and Biochemical Modifications after Laser Treatment

Previous research has suggested that stem cell bioactivity, such as cell migration, proliferation, survival, and overall cell niche, can be enhanced or positively modulated by PBM [11]. However, for these properties to be translated in clinical applications, more research is needed to define the best parameters of laser application and understand the underlying mechanisms of how PBM affects stem cell differentiation and proliferation. A study found that PBM LED irradiation treatment significantly increased the expression of SOX-9 at 830 nm and 10 J/cm^2^ [19], whilst the expression of CD34 was not significantly increased. One way that stem cell differentiation is thought to be enhanced by PBM is through the increase of ATP production, as glycolysis is known to be responsible for half of the energy required for osteogenic maturation [18]. However, as promising as this may sound, other studies, where a comparison of stem cell characteristics has been performed before and after laser treatment, have not observed any change in these cells’ activity. Mvula and Abrahamse suggested that the use of a low intensity laser irradiation (LILI) light source, combined with growth factors, improved the differentiation of adipose derived stem cells (ASCs) and confirmed their results through flow cytometry [25]. In the application of PBM on mesenchymal stem cells in naked mice models, Kim et al. showed that the ASCs population was larger with the use of laser therapy than in the controls, suggesting an increase in proliferation. Through studying protein expression levels, such as Ki67 and caspase 3, they found that the survival rate of the ASCs was also improved by the use of PBM [26]. Furthermore, growth factors and a higher expression of genes related to growth, like VEGF and bFGF, were observed in the target area compared to the control. This suggests that PBM improves the efficiency of the stem cells.

In a study conducted by Soleimani et al., the effect of different laser densities on bone marrow-derived stem cells using PBM was analyzed [27]. A range of energy density levels were investigated in BMSCs differentiation. The groups were previously induced to be either osteoblast or neural via cultivation in different media, and the groups are summarized in Table 1. They observed the osteogenic activity of stem cells by monitoring the levels of alkaline phosphatase (ALP), which is a by-product of formed osteoblasts. Interestingly, it was also suggested that the differentiation of the stem cells was dose-dependent as 4 J/cm^2^ culture groups were suggested to contain more osteoblasts than the 2 J/cm^2^ culture groups since the second day of culture. Furthermore, the 6 J/cm^2^ group had more than double the neurons than the 3 J/cm^2^ group. Although most papers are in agreement that 1–5 J is the optimal power density for proliferation, the study conducted by Migliario et al. found that the proliferation was highest at the range of of 5–50 J, peaking at 10 J, which is much higher [23]. However, their results at 50 J did suggest ROS had a toxic effect on the surrounding cells and saw a decrease in proliferation levels at that point. This emphasizes the importance of the other parameters involved in laser therapy which must be considered for the optimization of proliferation and differentiation rather than energy density alone. Furthermore, parameters for optimization vary for the target tissue types (skin or bone) [9], and should be accommodated in clinical applications.

**Figure 2:**
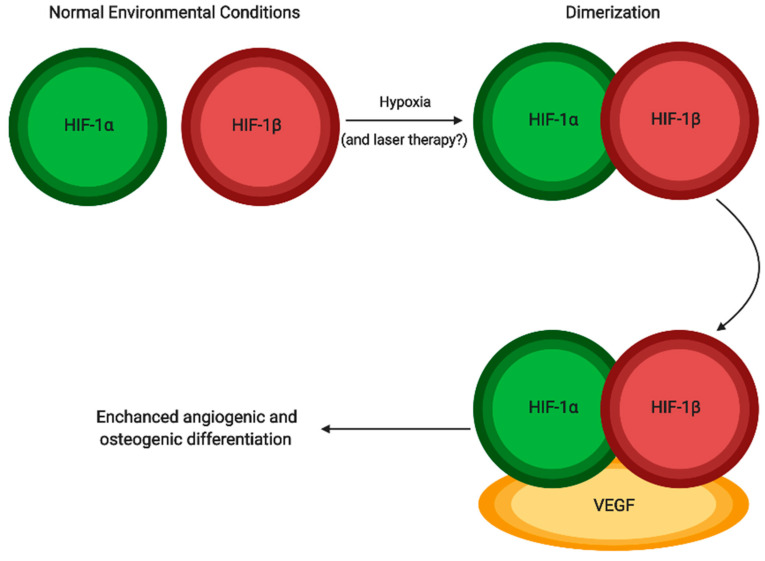
In hypoxic conditions, HIF-α undergoes dimerization with HIF-β. Laser therapy could possibly induce this affect further. The dimer then binds to VEGF; therefore, the gene expression is promoted and leads to angiogenesis and osteogenic differentiation. Created with BioRender.

To further understand its molecular mechanisms, Son et al. carried out a study to investigate how laser treatment effects cytokines and hormones under oxidative stress conditions [28]. The hormone melatonin was selected for interest due to their previous study suggesting that in hypoxic conditions, it stimulated cell differentiation with just 50 μM of the hormone used. Therefore, there was a control, a cell culture group with 50 μM of melatonin added, a group treated with laser therapy, and lastly a group treated with both melatonin and laser therapy. They also studied the effects of energy density on their laser treated samples, using a variety of values, such as 1.2, 2.4, and 3.6 J/cm^2^. Another important cellular mechanism that acts to combat oxidative stress and restore a normal cellular environment is the transcription factor HIF-1α (Figure 2) [28].

**Figure 3:**
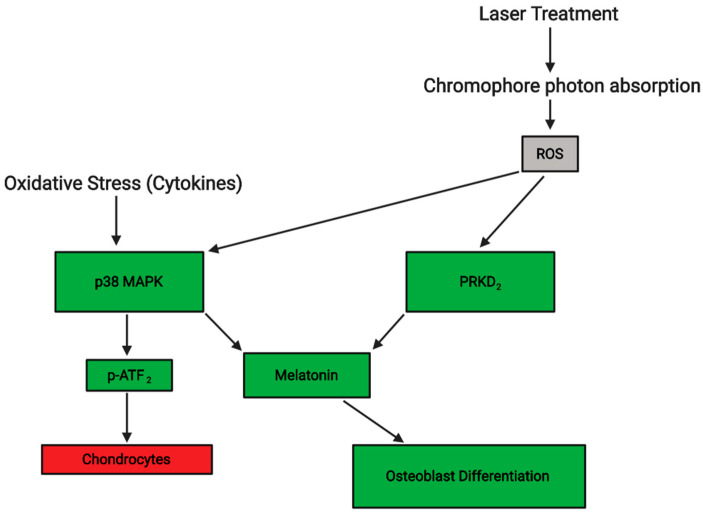
In the application of PBM, photon are absorbed by chromophores which activates ROS. As a secondary messenger, ROS results in the activation and stimulation of various pathways including p38 MAPK and PRKD_2_. Oxidative stress can also stimulate p38 MAPK by the presence of cytokines. The two pathways stimulate the hormone activity of melatonin, which induces osteoblast differentiation. However, p38 MAPK also increases p-ATF_2_ levels, which can result in decline of chondrocytes. Created with BioRender.

The transcription factor is stabilized in oxidative conditions and dimerization subsequently occurs with HIF-α and HIF-β, and, among other things, binds to *VEGF* and coincides with the increase of the gene’s expression in osteoblast cells. Bone healing is improved by laser therapies accelerating stem cell differentiation into osteoblasts and bone cells, and also through increasing calcium transport during the formation of bones [28]. Angiogenesis is also promoted by the influence of PBM on the regulation of VEGF and HIF-1α. RUNX2 is another transcription factor that is essential for osteoblast differentiation, and Osx acts downstream of it. PRKD_1_ increases the expression of Osx, as well as being involved in osteogenic differentiation by itself. Compared to the cells only treated with melatonin, in the study of Son et al. [28], the cells which had both the hormone and laser treatment had a higher level of ALP activity, and a higher percentage of bone mineralization by ~1.5%. Furthermore, the irradiated cell and melatonin cell combination group were still producing more ALP, whilst the other groups had peaked on day 14. In fact, the formation of mineralized bone was noticeably greater compared to the non-irradiated cells after one week. At two weeks, the number of Alizarin Red-stained cells were at least two-fold that in the control group, which suggests an increase in calcium deposits laser treated cells. The authors suggested that the pathway p38 MAPK is activated under stressful stimuli like cytokines and responds by encouraging the differentiation of cells. In the oxidative stress conditions, the pathways of PRKD_1_ and p38 MAPK are suggested to be the primary signaling molecules for regulating the osteoblastic differentiation actions of melatonin [29]. Both of these pathways’ activities are stimulated by ROS, and thus by laser treatment as seen in Figure 3. To establish if these pathways are responsible for the increased osteogenic proliferation and differentiation, and if there are other pathways involved such as ones that increase adipose stem cell activity, more research is required.

## 4. Tissue Regeneration after Laser Treatment

In cases of severe bone loss, the tissue engineering approach for bone regeneration might incorporate implementation of scaffold, growth factors and stem cells to enhance an influx of osteogenic cells to the targeted area and encourage the body’s natural repair/healing capability [30]. The application of laser treatment, which enhances both differentiation and proliferation of stem cells, should therefore positively contribute. In an experiment conducted by Abramovitch-Gottlib et al. mesenchymal stem cells differentiation into bone cells was investigated where cells were cultured in a 3D system and PBM was applied [31]. In this study, osteogenic-like cell production was more present in their irradiated (treated) group of cells compared to the control, resulting in more differentiated cells. This provides further evidence that PBM enhanced mesenchymal differentiation into osteogenic cells. The positive effects of PBM were also suggested when observing the healing process of post-surgical bone grafts, in cases such as fractures and bone reconstruction, highlighting a higher healing rate and avoiding infections [32,33]. In addition, PBM enhancing bone regeneration could expand the scope of clinical strategies in cases with bad prognosis such as spinal cord injuries [34]. However, there is also a study with results that disagree with this optimistic belief and found no significant improvement in postoperative dogs treated with PBM therapy for intervertebral disk disease [35].

Another approach by which laser therapy can aid the process of tissue repair and regeneration is by increasing bone grafts porosity. Bone grafts must provide conditions for new blood vessels to be formed and in order to do so, bone grafts must have certain porosity [36]. Larger damaged areas of bone that need to be repaired are correlated to an increased likelihood of failing, due to the need of a larger blood supply [37,38]. After studying laser irradiation potential in producing pores, Sobol et al. suggested that micropore production may be improved through the use of lasers [39]. To create the pores, the irradiation applied to the cartilage was a wavelength of 1560 nm, power output of 0.9 W and with a pulse duration of 100 ms.The process of forming pores starts in an area of expanding heat that reaches cold areas, and this causes local strains and disruption of homeostasis. Pores are then made as a mechanism to cushion the mechanical stress caused by this [40], and the technique of pulse repetitive laser radiation induces porous activity by introducing repetitive heat in the tissue [41]. Mesenchymal stem cells treated by PBM can be potentially used in liver repair and enhanced by stimulating angiogenesis. A study conducted by Uri Oron and collaborators [42], using mature rat livers, suggested that the group treated with 804 nm light had 2.6-fold greater proliferating cell number than the non-laser treated control group. Furthermore, the cell numbers in the newly made blood vessels and the immunopositive stem cells were respectively greater than the control by 3.3- and 2.3-fold.

PBM has also shown to be beneficial in other clinical applications. Skin is the most susceptible tissue to laser, due to its role of acting as a barrier to protect the body from external factors and therefore a good candidate to experiment with laser therapy [9]. In a study focusing on the effectiveness of laser therapy on healing burn wounds, Rathnakar et al. observed various parameters on healing the burns in mice [43]. Out of the wavelengths 632.8 nm, 785 nm, and 830 nm, the largest wavelength was shown to be the most effective. The power density of the lasers was then tested with 830 nm wavelength, and they found optimum results with 3 J/cm^2^ performing best in proliferation and resulting in healing process. In wound healing, the study also concluded that 830 nm combined with 3 or 4 J/cm^2^ was similar to 632.8 nm combined with 2 J/cm^2^, but the former still performed significantly faster in healing. A different study looked at the use of lasers in weight loss, suggesting that weight can be lost through the application of lasers, possibly due to the creation of pores that leaked triglycerides [17].

## 5. Types of Lasers and Their Application in Medicine and Dentistry

In the last decade, we have observed an increase in the number of studies describing the use of laser therapy in medicine and dentistry [44,45]. Lasers with different characteristics have been employed in different branches of medicine and dentistry. With wavelengths of 445–980 nm, diode lasers consists of a system of many semiconductors designated as conductor bands or valence bands, between which the flow of electric current causes the excitation of electrons and the creation of a laser wave [46,47,48]. Depending on the composition of semiconductors, a wave with different frequency (length) can be generated. Some of the most popular lasers in implant surgery for cutting soft tissues are lasers with waves in the range of 808–980 nm. In turn, to stimulate wound healing after surgery, we can use diode lasers with wavelengths in the range of red light (630–635nm) or near-infrared (780–808nm). The effectiveness of semiconductor lasers in soft tissues is associated with their high absorption in hemoglobin and melanin and with deep penetration of the beam in tissues in the range of 5–10 mm [49,50]. Erbium-YAG laser, 2940 nm, shows great absorption in hydroxyapatite and in water, translating into high cutting efficiency, low dispersion, and low tissue penetration in the range of several dozen micrometers. The medium of the laser contains an erbium crystal with an admixture of yttrium, aluminum, and garnet. Inversion of a population in the Er:YAG laser is done by pumping the system with a xenon lamp [51,52,53,54,55]. Er,Cr:YSGG produces a wavelength of approximately 2780 nm, well absorbed in water and hydroxyapatite. The laser resonator contains a crystal of erbium and chromium with a mixture of yttrium, scandium, gallium, and garnet. The Er,Cr:YSGG laser is characterized by low penetration in tissues, which amounts to several dozen micrometers [55]. A CO_2_ laser was constructed in 1964 by Patel [53]. The active medium in the resonator system contains carbon dioxide, nitrogen, hydrogen, xenon, and helium [55]. Depending on the design, the laser generates waves with a length of 9300, 9600, or 10,600 nm, which are best absorbed by hydroxyapatite and well absorbed by water. The depth of penetration of tissues is from 0.1 to 0.23 mm [55]. Neodymium-YAG laser produces a beam with a wavelength of 1064 nm, which is well absorbed by hemoglobin and melanin. The active crystal contains neodymium with the addition of yttrium, aluminum, and garnet. Mainly used for vaporization of soft tissues and also for photobiomodulation. The laser produces pulses in nanosecond width. This laser is characterized by deep penetration in tissues [43,49].

As previously mentioned, the important role of laser irradiation is the induction of PBM, used to accelerate the healing of various tissues and increase the number of cells and their vitality [56,57]. The photobiomodulatory effect induced mainly by the diode lasers with wavelengths in the red and near-infrared range (630–940 nm) affects a modulation of cell proliferation. The use of lasers with wavelengths from an “optical window” (600–1100 nm) result in deeper penetration and therefore evokes a wider cell-light response [58,59]. The Arndt–Schultz’s curve is used to describe the dose-dependent effects of PBM and it suggests that a weak stimulus increases physiologic activity, while moderate stimuli inhibit activity, and in the case of extreme stimuli, the activity is eliminated [58,59]. That highlights the importance of the appropriate and monitored dosage. It has been shown that the utilization of fluence in the range of 1–5 J/cm^2^ is optimal to receive an optimal biological response [58]. PBM involves the application of a monochromatic light with a low energy density which induces non-thermal photochemistry effects on cellular level [60]. This method has been proposed as innovative, enabling enhancement of the process of bone healing and at the same time increasing primary stability [60]. In the field of dentistry, several studies have documented an increase in the stability of implants and bone-implant contact (BIC) factor after implant laser light [61,62]. PBM with low-energy density range was shown to stimulate the mitochondrial and cellular membrane photoreceptors to synthesize ATP, which enhanced cell proliferation rate [49,63]. A biostimulatory effect on bone tissue was also reported by increasing proliferation and differentiation of osteoblasts, cells responsible for bone formation [50]. This is in accordance with the results of Al Ghamdi et al. [58] who reported that PBM can induce mitosis in cultured cells, collagen production, and DNA and RNA synthesis. Several studies showed the effects of PBM in the healing process, focusing on soft and hard tissue repair following surgeries, where the healing process significantly improved [64,65]. This “revitalizing” process as a result of enchanced healing is followed by nerve regeneration as reported by Mohammed et al. [50].

The important advantages of PBM usage in promoting the proliferation of human fibroblasts when using appropriate parameters were reported by different research groups [66,67]. Kreisler et al. [64] examined the effects of the low-level diode (809 nm) laser irradiation on the proliferation rate of human gingival fibroblasts (HGF) in vitro. The HGF cells were irradiated alternatively once, twice, and three times at a 24-hour interval at energy fluences of 1.96–7.84 J/cm^2^ and exposure time between 75 and 300 s. The authors reported improvement in HGF’s proliferation activity 24-hour post-irradiation (*P* < 0.05) and its reduction in an energy-dependent manner 48 and 72 h after the laser treatment. Laser-induced cellular outcomes were similar within the range of 2–8 J/cm^2^. Khadra et al. [7] tested the effect of PBM on the attachment and proliferation of HGFs grown on the titanium implant surface. The HGFs were irradiated using an 830-nm diode laser for three consecutive days at dosages of 1.5 or 3 J/cm^2^, and left to grow for eight and 10 days. It was concluded that PBM with the diode laser at a fluence of 1.5 and 3 J/cm^2^ enhances the attachment and proliferation of HGF on titanium discs. Almeida-Lopes et al. [66] also analyzed the effect of PBM on the human gingival fibroblasts. They applied a diode laser with the various wavelengths: 670 nm, 780 nm, 692 nm, and 786 nm at the same energy density of 2 J/cm^2^. The evaluation periods were two, four, and six days after the laser application. The authors concluded that PBM had improved the in vitro fibroblast proliferation, and a smaller laser exposure time results in a higher proliferation of HGF.

PBM has been widely used for the therapy of numerous diseases like candidiasis treatment [54], periodontitis [51,68,69,70,71]. PBM was reported to improve the effect of different medical therapies when applied in conjunction with the therapies.

### 5.1. PBM in Diabetes Treatments

Diabetes is a common disease and a global public health problem [72]. Studies on PBM application for diabetic patients showed that PBM could reduce insulin level by almost three-fourths or allows for discontinuing medication for six months for Type 1 and Type 2 diabetes, respectively [73]. PBM treatment is advantageous in treating diabetes, as evidenced by knowledge gained with its use over many years. PBM seems to have antioxidant and immunomodulating effects that improve microcirculation and myocardial contractility [72,73].

### 5.2. PBM Application in Neural Diseases Treatments

Transcranial PBM improves regional cerebral blood flow in individuals with traumatic brain injuries, as well as in severe depression and Parkinson’s disease [74,75]. A rise in ATP production and improved regional cerebral blood flow are essential factors that could provide faster repair of the affected nervous system. It was suggested that NIR therapy saves many dopaminergic cells from cell death. This effect of PBM was utilized in the treatment of Parkinson s Disease [75]. Naeser et al. [76] described two case reports for chronic brain injury where cognition improved following PBM treatment. The patients reported improved sleep and better capability to perform social, interpersonal, and occupational functions [76]. Furthermore, Azbel et al. [75] reported the earliest study on a brain animal model in 1993. The authors described the improvement of synaptic conductance in hippocampal neurons after PBM.

PBM has also been used for spinal cord repair. The spinal cord is a delicate, long, tail-shaped structure that starts at the end of the brain stem and stretches forward to the end of the spine. The spinal cord consists of nerves that transport signals between the brain and the other parts of the body. A positive effect of PBM for spinal cord repair (SCI) was described by Huang et al. [77]. Their findings suggest the effectiveness of intravascular laser irradiation to blood in alleviating oxidative stress and mitochondrial dysfunction in chronic SCI patients [77]. In turn, Yamany et al. [78] implemented PBM to thirty subjects with painful diabetic neuropathy and obtained a pain reduction, change of foot skin microcirculation, and some electrophysiological parameters of peripheral nerve function.

### 5.3. PBM in Dermatology

The effectiveness of PBM was described and proven in various dermatological therapies, including skin rejuvenation, hair loss treatment, and fat loss procedures.

The efficiency of PBM was utilized for improving skin conditions such as wrinkles and skin laxity [79]. Wikramanayake et al. [80] confirmed the hair growth results of PBM on the C3H/HeJ mouse model using a 655-nm laser wavelength for 20 s three times per week for a total of six weeks.

### 5.4. PBM in Management of Secondary Complications Following Radiation

Fungal mucositis often appears as a consequence of oral radiation therapy and significantly impairs the quality of life of patients. The efficient management of this complication is consequently crucial. However, there is an insufficiency of randomized clinical studies of oral care concerning this problem [81].

Ciais et al. [82] in 1985 described the effectiveness of PBM with 10–150 mW He/Ne or diode laser by decreasing the severity of oral mucositis lesions in vivo. The effectiveness of PBM in the prevention of chemotherapy-induced oral mucositis was also highlighted in a randomized clinical trial conducted by Cowen et al. [83] in patients undergoing bone marrow transplantation. Officially, the application of PBM was recommended by The Multinational Association of Supportive Care in Cancer (MASCC) and the International Society of Oral Oncology (ISOO) in 2014 for patients undergoing hematopoietic stem cell transplantation high-dose chemotherapy [84].

### 5.5. PBM in Dentistry

Laser and light-emitting diodes (LEDs) are being used in almost every field of clinical dentistry, changing the dental healthcare approaches and patient’s quality of life. Dental applications for PBM are based on usage of low dose of biophotonics therapy. PBM in orthodontic treatment is used to reduce pain after orthodontic appliance placement [85,86]. It also improves osseointegration, collagen deposition, and achieves faster bone-remodeling [87,88,89]. In surgical-assisted therapy, such as implant and mini-implant placement, it was shown to assist implant stability [57], healing time was reduced, along with swelling, contributing to an improvement of postoperative comfort [88,89]. In the literature, a significant number of scientific papers have reported advantages of using laser light stimulation in oral medicine, in clinical situations as recurrent aphthous stomatitis, herpes infections, mucositis, and burning mouth syndrome [90,91,92]. Recurrence aphthous stomatitis (RAS), also known as the recurrent oral ulcer, is a most common oral lesion that can be classified as a minor, major, or herpetiform ulcers. Although the cause of oral lesion formation is not entirely known, it is related to immune system dysfunction, genetic factors, allergic agents, nutrition, hormonal changes, stress, and infective viruses. Clinically it can be manifested as small, round or ovoid, painful, self-healing, and recurrent ulcers with circumscribed margins, erythematous haloes, and yellow or gray floors [92]. Jijin et al. [91] reported that low level diodide laser therapy, compared to of 5% Amlexanox oral paste, a commonly used treatment, presented a statistically significant reduction in pain score after three days, although no difference was observed after day seven. Tezel et al. [92] reported that the Nd:YAG laser at 100 mJ, 2 W, 20 Hz for 2–3 min in contact mode has better patient acceptance, shorter treatment time, and lower rates of pain. Recently Han et al. [90] reported significantly alleviated pain (especially the immediate pain relief) with facilitated healing compared with a placebo group. PBM in herpes infections treated at doses below 10 J/cm^2^ has been shown to have positive effects in pain reduction, accelerating the healing process, as well as contributing to viral resistance mechanism inhibition and recurrence reduction [93,94,95]. A multicenter randomized, double-blind controlled trial in oncologic children showed that laser therapy changed the grade of oral mucositis significantly and decreased pain score [96]. Eliminating pain is a major interes, especially in pediatric patients. Often assessing pain in young children is challenging, taking into consideration the differences of pain perception of different age groups. The risk to benefit ratio is particularly favorable to PBM as it contributes to a reduction in hospitalization days, and thus costs, as well as am improvement of the phonatory, swallowing, and chewing capacity [96]. PBM in the NIR (980 nm) with fluence of 4 J/cm^2^ was described as a useful treatment for oral lichen planum indicating pain and injury reduction compared to the control group [97].

The application of PBM has been recognized as an adjunctive or alternative approach in periodontal and peri-implant inflammation therapy [68,69,98,99,100].

Although the biostimulatory effect of lasers is well-established and defined by different parameters, such as wavelength, energy density, power output, and duration of radiation, further research on the possibilities of stem cell modulation, addressing the healing process, is in need of investigation, specifically considering the significant results obtained by applying stem cells and laser treatment in conjunction with other already established clinical therapies.

The need for translating the acquired knowledge of the underlying mechanisms of PBM, the properties of different stem cells, and the mechanisms of repair and regeneration should be joined and translated into novel therapeutic approaches.

## Figures and Tables

**Table 1:** The effect of different laser densities on bone marrow-derived stem cells.

Culture Group	Energy Density (J/cm^2^)	Enhanced Proliferation and Differentiation Induced Cell Type
1	2	Osteoblast
2	3	Neural
3	4	Osteoblast
4	6	Neural

The four different BMSCs culture groups used. Groups 1 and 3 were cultured to be induced to differentiate into osteoblasts; groups 2 and 4 were cultured to be induced to differentiate into neurons. The middle column states the energy density of the laser used [27].
